# XIAP deletion sensitizes mice to TNF-induced and RIP1-mediated death

**DOI:** 10.1038/s41419-023-05793-1

**Published:** 2023-04-11

**Authors:** Axel Witt, Tatiana Goncharov, Yujung Michelle Lee, Matthias Kist, Monika Dohse, Jeff Eastham, Debra Dugger, Kim Newton, Joshua D. Webster, Domagoj Vucic

**Affiliations:** 1 Department of Immunology Discovery, Genentech, South San Francisco, CA 94080 USA; 2 Department of Pathology, Genentech, South San Francisco, CA 94080 USA; 3 Department of Physiological Chemistry, Genentech, South San Francisco, CA 94080 USA; 4 Present Address: Neovii Pharmaceutical AG, 8640 Rapperswil, Switzerland; 5 Present Address: CatalYm GmbH, Am Klopferspitz 19, 82152 Munich, Germany

**Keywords:** Sepsis, Apoptosis

## Abstract

XIAP is a caspase-inhibitory protein that blocks several cell death pathways, and mediates proper activation of inflammatory NOD2-RIP2 signaling. XIAP deficiency in patients with inflammatory diseases such as Crohn’s disease, or those needing allogeneic hematopoietic cell transplantation, is associated with a worse prognosis. In this study, we show that XIAP absence sensitizes cells and mice to LPS- and TNF-mediated cell death without affecting LPS- or TNF-induced NF-κB and MAPK signaling. In XIAP deficient mice, RIP1 inhibition effectively blocks TNF-stimulated cell death, hypothermia, lethality, cytokine/chemokine release, intestinal tissue damage and granulocyte migration. By contrast, inhibition of the related kinase RIP2 does not affect TNF-stimulated events, suggesting a lack of involvement for the RIP2-NOD2 signaling pathway. Overall, our data indicate that in XIAP’s absence RIP1 is a critical component of TNF-mediated inflammation, suggesting that RIP1 inhibition could be an attractive option for patients with XIAP deficiency.

## Introduction

X-chromosome-linked inhibitor of apoptosis (XIAP) is an IAP protein that controls cell death induced by a variety of stimuli and mediates inflammatory signaling. XIAP was originally identified as a caspase-inhibitory protein that can block extrinsic and intrinsic apoptotic cell death [1]. However, XIAP also plays important roles in tumor necrosis factor (TNF)- or lipopolysaccharide (LPS)-stimulated necroptosis and inflammatory cell death [2–4]. In particular, in the absence of XIAP, LPS stimulation leads to RIP3-dependent cell death and release of IL-1β [3, 5, 6]. In addition, XIAP is a critical mediator of NOD2-RIP2 signaling [7]. Activation of NOD2 leads to RIP2 recruitment and engagement of XIAP to promote K63-linked and linear RIP2 ubiquitination [8]. These posttranslational modifications enable activation of NF-κB and MAPK signaling, and the production of multiple inflammatory cytokines and chemokines [7–9]. Preventing XIAP-RIP2 interactions with either IAP selective antagonist XB2m54 or the RIP2 kinase inhibitor GSK583 blocks XIAP-mediated RIP2 ubiquitination and activation of inflammatory signaling [9]. *NOD2* mutations are associated with Crohn’s disease [10, 11], suggesting that aberrant NOD2-RIP2-XIAP signaling may contribute to inflammatory bowel disease (IBD).

*XIAP* is mutated in several inflammatory diseases, including X-linked lymphoproliferative syndrome type II (XLP-2) [12, 13]. XLP-2-associated mutations are found throughout the XIAP protein, and affected boys often present with early onset inflammatory symptoms [13–16]. For patients needing hematopoietic cell transplantation (HCT), XIAP deficiency can promote graft-versus-host disease (GVHD) driven by donor T cell activation [17]. Indeed, XIAP deficiency is regarded as a high risk for allogeneic HCT and leads to suboptimal outcomes [16, 18]. Loss-of-function *XIAP* mutations have also been reported in male patients with early-onset Crohn’s disease [19, 20]. Typically, Crohn’s disease in patients with XIAP deficiency is severe and difficult to treat [15, 21]. The intestinal microbiota has been shown to contribute to intestinal inflammation when XIAP is absent [22, 23].

Given the strong genetic link between XIAP deficiency and inflammatory diseases, including Crohn’s disease, plus poor clinical outcomes for patients bearing deleterious XIAP mutations, we explored inhibition of RIP1-dependent cell death as a therapeutic option. We show that XIAP loss sensitizes cells and mice to LPS- and TNF-induced cell death, while sparing NF-κB and MAPK signaling. Furthermore, we demonstrate that RIP1 inhibition effectively blocks TNF-stimulated cell death, hypothermia, lethality, cytokine and chemokine release, intestinal tissue damage and granulocyte migration in XIAP deficient mice. Inhibition of the related kinase RIP2 did not affect TNF stimulated events, suggesting a lack of involvement for the RIP2-NOD2 signaling pathway. Our data indicate that the kinase activity of RIP1 is a critical for TNF-mediated inflammation in XIAP-deficient mice. Therefore, it is worth exploring whether XIAP-deficient patients might benefit from RIP1 inhibition.

## Results

### XIAP deficiency enhances LPS- and TNF-mediated cell death

**Fig. 1:**
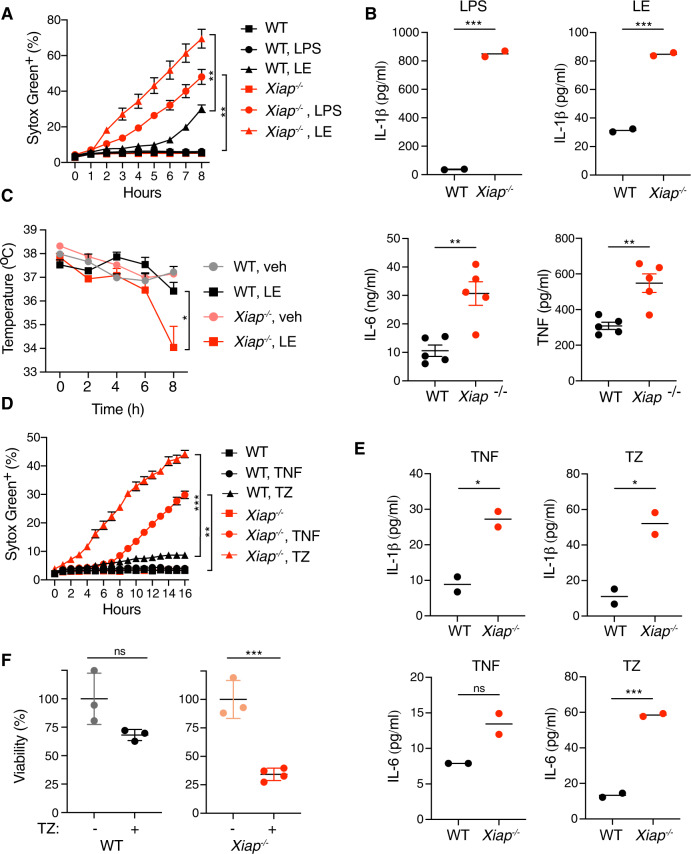
XIAP deficiency enhances LPS and TNF mediated cell death. **A** BMDMs derived from WT or *Xiap*^*−/−*^ mice were treated with LPS or LPS and Emricasan (LE). Percentage of dead BMDMs (Sytox Green positive cells) compared to positive control. Signal was measured every hour in the Incucyte with the indicated treatments: LPS 100 ng/ml, Emricasan 5 μM. (*n* = 5 for WT non-treated control, *n* = 6 for all others). **B** IL-1β release from indicated BMDMs (*n* = 2) treated with 100 ng/ml LPS or LPS and 5 μM Emricasan (LE) for 8 h. **C** LPS and Emricasan (LE) induced hypothermia (left), WT (*n* = 5 mice for vehicle and *n* = 15 for LE) and *Xiap*^*−/−*^ (*n* = 5 mice for vehicle and *n* = 18 for LE) and release of IL-6 and TNF at 8 h (right), WT (*n* = 5) and *Xiap*^*−/−*^ (*n* = 5) mice. **D** BMDMs derived from WT or *Xiap*^*−/−*^ mice were treated with TNF (50 ng/ml) or TNF and zVAD (20 μM) (*n* = 6 for each treatment). Cell death was performed as described for **A**. **E** IL-1β and IL-6 release from indicated BMDMs (*n* = 2) treated with 50 ng/ml TNF or TNF and zVAD (TZ) for 8 h. **F** Intestinal organoids derived from WT or *Xiap*^*−/−*^ mice were treated with TNF and zVAD (TZ) (for *Xiap*^*−/−*^ TZ treatment *n* = 4, for all other treatments *n* = 3 mice). Viability was determined 24 h later using MTT assay. In **A**, **C**, **D**, bars indicate median with standard error, in **B**, **E** median, in **F** mean with standard deviation. Ns indicates no significance, *p < 0.05, **p < 0.01, ***p < 0.005.

We investigated the role of XIAP in LPS- or TNF-mediated cell death using bone marrow-derived macrophages (BMDMs) from wild-type (WT) or *Xiap* knockout (*Xiap*^*−/−*^) mice. Compared to WT BMDMs, *Xiap*^*−/−*^ BMDMs exhibited increased cell death in response to LPS, LPS plus pan-caspase inhibitor emricasan (LE), or LPS plus pan-caspase inhibitor zVAD (LZ) (Figs. 1A and S1A). LPS alone, and to a lesser extent LE, promoted the release of IL-1β from *Xiap*^*−/−*^ BMDMs, but not WT BMDMs (Fig. 1B). LPS also induced processing of caspases 3, 7 and 8 in *Xiap*^*−/−*^ BMDMs, which was reduced by the addition of emricasan (Fig. S1B). As expected, caspase inhibition promoted RIP3 and MLKL phosphorylation, hallmarks of necroptosis signaling (Fig. S1B). Loss of XIAP did not alter LPS-induced NF-κB and MAPK signaling (Fig. S1C). *Xiap*^*−/−*^ mice treated with LE exhibited more severe hypothermia than WT mice at 8 h after dosing, and this correlated with increased serum IL-6 and TNF (Fig. 1C). *Xiap*^*−/−*^ mice were also more susceptible than WT mice to liver damage induced by LPS plus the transcriptional inhibitor D-galactosamine (GalN), exhibiting elevated serum AST and ALT at 5 h after treatment (Fig. S1D). These results indicate that loss of XIAP sensitizes BMDMs and mice to LPS-mediated cell death and tissue damage.

Treatment with TNF alone, or in combination with zVAD (TZ), also induced more cell death in *Xiap*^*−/−*^ BMDMs than WT BMDMs, and the XIAP-deficient cells released more IL-6 and IL-1β (Fig. 1D, E). Intestinal organoids derived from *Xiap*^*−/−*^ mice also exhibited more cell death in response to TZ than WT organoids (Fig. 1F). Nevertheless, TNF induced NF-κB and MAPK signaling was normal in *Xiap*^*−/−*^ BMDMs (Fig. S1E). Thus, altered NF-κB and MAPK signaling is not responsible for the enhanced sensitivity of XIAP-deficient BMDMs and organoids to TNF-mediated cell death.

### XIAP deletion sensitizes mice to TNF toxicity

**Fig. 2:**
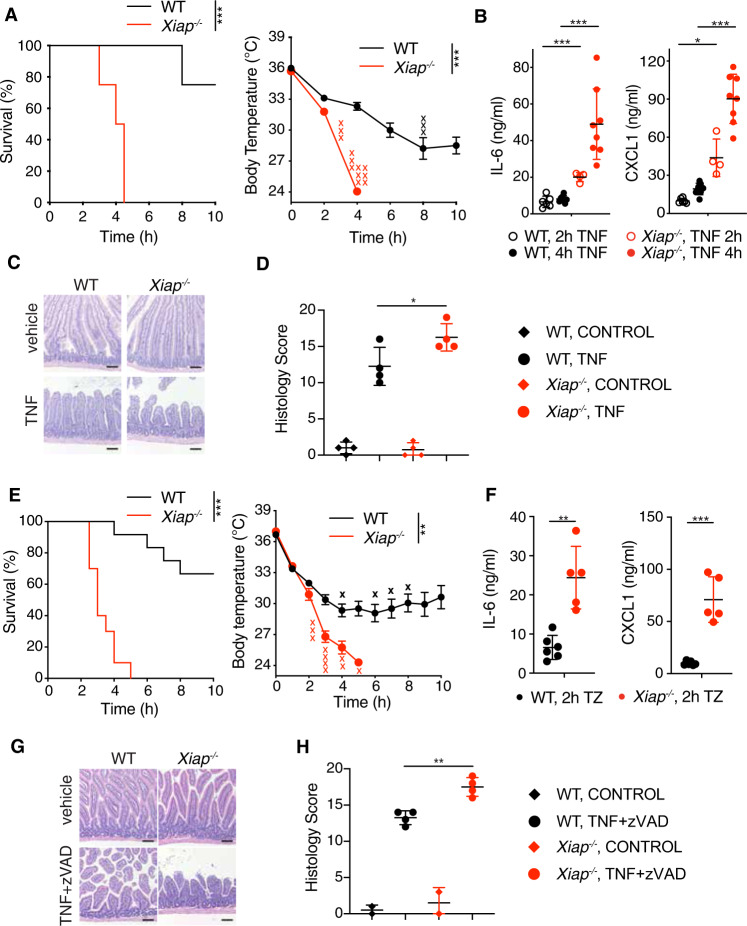
XIAP deletion sensitizes mice to TNF induced lethality in vivo. **A** WT (*n* = 12) and *Xiap*^*−/−*^ (*n* = 12) mice were injected with TNF (500 μg/kg). Survival (left) and body temperature (right) were monitored for 10 h. X indicates animals that had to be euthanized. **B** Serum levels of indicated cytokines from WT (*n* = 6 mice for 2 h and *n* = 8 for 4 h treatment) and *Xiap*^*−/−*^ mice (*n* = 4 mice for 2 h and *n* = 8 for 4 h treatment) post TNF injection were analyzed by Luminex. **C**, **D** Hematoxylin and eosin-stained small intestines of WT and *XIAP*^*−/−*^ mice 4 h post TNF injection (**C**) and quantification of the histology score (**D**) (*n* = 4 mice per each condition). Size bar = 100 μm. **E** WT (*n* = 12) and *Xiap*^*−/−*^ (*n* = 10) mice were injected with TNF (300 μg/kg) and zVAD-FMK (10 mg/kg). Survival (left) and body temperature (right) were monitored for 10 h. X indicates animals that had to be euthanized. **F** Serum levels of indicated cytokines from WT (*n* = 6) and *Xiap*^*−/−*^ (*n* = 5) mice 3 h post TNF and zVAD (TZ) injection were analyzed by Luminex. **G**, **H** Hematoxylin and eosin-stained small intestines of WT and *Xiap*^*−/−*^ mice 3 h post TNF and zVAD injection (**G**) and quantification of the histology score (**H**) (*n* = 2 mice per control groups and *n* = 4 mice for treatment). Size bar = 100 μm. In **A**, **E**, bars indicate median with standard error, in **B**, **D**, **F**, **H** mean with standard deviation. *p < 0.05, **p < 0.01, ***p < 0.005.

*Xiap*^*−/−*^ mice were also more sensitive than WT mice to high dose TNF or TZ, exhibiting more severe hypothermia, enhanced production of serum IL-6 and CXCL1, increased intestinal damage, and more animals had to be euthanized (Fig. 2A–H). Consistent with this genetic experiment, TNF toxicity in WT mice was exacerbated by the XIAP selective antagonist XB2m54 [9] (Fig. S2A, B), albeit XB2m54 did not have as dramatic an effect as XIAP deficiency. Importantly, XB2m54 did not affect c-IAP1/2 levels in mice (loss of c-IAP1/2 is a readout for c-IAP1/2 antagonism [24]), thus confirming that compound’s effect is limited to XIAP (Fig. S2C). XB2m54 also enhanced TZ-induced hypothermia in WT mice (Fig. S2D).

### XIAP deletion sensitizes cells and mice to RIP1-dependent TNF-induced lethality and intestinal damage

**Fig. 3:**
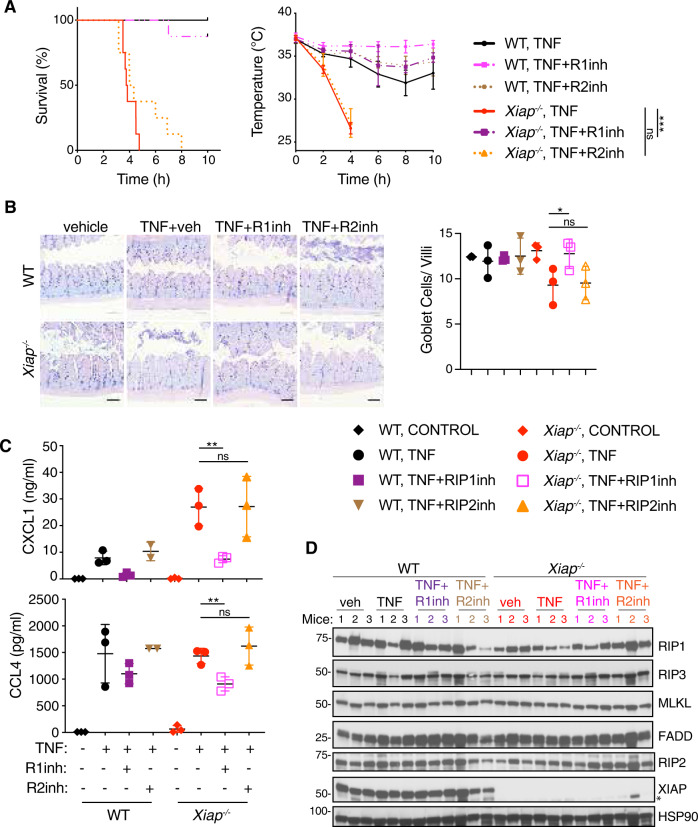
XIAP deletion sensitizes mice to RIP1-dependent TNF-induced lethality and intestinal damage. WT and *Xiap*^*−/−*^ mice were given vehicle or TNF (500 μg/kg) in the absence or presence of RIP1 inhibitor GNE684 (50 mg/kg) or RIP2 inhibitor GSK583 (30 mg/kg). **A** TNF induced lethality (left) and hypothermia (right) in WT and *Xiap*^*−/−*^ mice treated with RIP1 inhibitor GNE684 or RIP2 inhibitor GSK583. (*n* = 7 mice for *Xiap*^*−/−*^ TNF + R1inh treatment, *n* = 8 mice for all other treatments). **B** RIP1 inhibition restores Goblet cells in TNF treated (2 h) *Xiap*^*−/−*^ mice. Representative images shown on the right and quantification on the left. Size bars = 100 μm. (*n* = 3 mice for each treatment). **C** RIP1 inhibitor GNE684 reduces the release of CXCL1 and CCL4 in TNF treated (2 h) *Xiap*^*−/−*^ mice. (*n* = 2 mice WT treated with TNF + R2inh, *n* = 3 for all other treatments). **D** Protein levels in small intestines of WT and *Xiap*^*−/−*^ mice treated with TNF (2 h) in the absence or presence of RIP1 or RIP2 inhibitors. Western blots were performed with indicated antibodies (*n* = 3 mice for each treatment). Asterisk indicates nonspecific band. In A, bars indicate median with standard error, and in **B**, **C** mean with standard deviation. Ns indicates no significance, *p < 0.05, **p < 0.01, ***p < 0.005.

We investigated the importance of the kinase activity of RIP1 in the enhanced response of *Xiap*^*−/−*^ BMDMs to TNF or TZ using the RIP1 inhibitor GNE684 [25] (Fig. S3A, B). The death of *Xiap*^*−/−*^ BMDMs after TNF or TZ treatment was reduced significantly by GNE684. By contrast, the RIP2 inhibitor GSK583, which can prevent XIAP-RIP2 association [9, 26], had no discernible effect on TNF- or TZ-induced death of WT or *Xiap*^*−/−*^ cells (Fig. S3A, B). Similarly, inhibition of RIP1, but not RIP2, suppressed TNF-induced morbidity and hypothermia in *Xiap*^*−/−*^ mice (Fig. 3A). Goblet cell loss, which was observed after TNF dosing in *Xiap*^*−/−*^ mice, but not WT mice, was also ameliorated by GNE684 (Fig. 3B). RIP1 inhibition also reduced levels of CXCL1 and CCL4 in the serum of *Xiap*^*−/−*^ mice (Fig. 3C). The lack of effect of RIP2 inhibition was not due to the absence of RIP2, because we detected comparable levels of RIP2 in WT and *Xiap*^*−/−*^ intestines (Fig. 3D). RIP1 inhibitor GNE684 also blocked LPS induced necroptotic cell death in *Xiap*^*−/−*^ BMDMs (Fig. S3C). Thus, in the absence of XIAP, TNF activates RIP1 to cause tissue damage and the release of cytokines and chemokines.

### XIAP deficiency promotes TNF-stimulated and RIP1-dependent granulocyte migration but does not affect TNF-induced gene expression

**Fig. 4:**
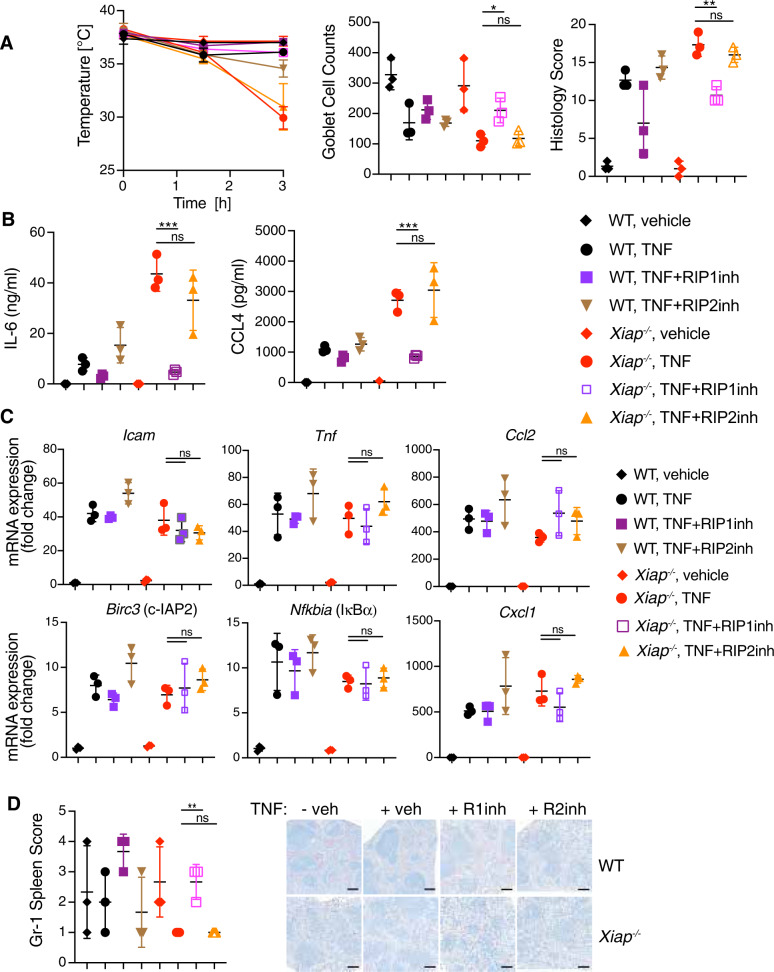
XIAP deficiency promotes TNF stimulated and RIP1 dependent granulocyte depletion from spleen but does not affect TNF induced gene expression. WT and *Xiap*^*−/−*^ mice were treated with vehicle or TNF (500 μg/kg) for 3 h in the absence or presence of RIP1 inhibitor GNE684 (50 mg/kg) or RIP2 inhibitor GSK583 (30 mg/kg). **A** TNF induced hypothermia (left), Goblet cell loss (middle) and intestinal damage (left) in WT and *Xiap*^*−/−*^ mice can be ameliorated by RIP1 inhibitor but not by RIP2 inhibitor. (*n* = 3 mice for each treatment). **B** RIP1 inhibitor reduces the release of IL-6 and CCL4 in TNF treated *Xiap*^*−/−*^ mice. (*n* = 3 mice for each treatment). **C** XIAP absence or RIP1 inhibition do not affect TNF stimulated gene expression. RT-qPCR of indicated genes using small intestines of WT (*n* = 3) or *Xiap*^*−/−*^ (*n* = 3) mice untreated or treated with TNF (3 h) in the absence or presence of RIP1 or RIP2 inhibitors. **D** RIP1 inhibition reduces the depletion of granulocytes from spleens of TNF treated *Xiap*^*−/−*^ mice. Gr-1 IHC was performed on sections of spleens from WT (*n* = 3) or *Xiap*^*−/−*^ (*n* = 3) mice treated with TNF (3 h) and given RIP1 or RIP2 inhibitors. Quantification of Gr-1 positive cells depicted in the graph (left) with representative images (right). Size bars = 200 μm. In panel A bars indicate mean with standard error for temperature and mean with standard deviation for goblet cells and histology in **A** and all graphs in **B**–**D**. Ns indicates no significance, *p < 0.05, **p < 0.01, ***p < 0.005.

Next, we examined the spleens and livers of an independent cohort of *Xiap*^*−/−*^ mice at 3 h after dosing with TNF. Consistent with our earlier results, *Xiap*^*−/−*^ mice displayed RIP1-dependent hypothermia, intestinal damage, goblet cell loss, and release of IL-6 and CCL4 (Fig. 4A, B). We investigated if loss of XIAP or inhibition of RIP1 affected TNF-induced gene expression. Small intestines from WT and *Xiap*^*−/−*^ mice expressed comparable amounts of *Icam, Tnf, Ccl2, Birc3, Nfkbia*, and *Cxcl1* mRNAs after TNF treatment, and this was not altered by RIP1 inhibition (Fig. 4C).

IL-6 and CCL3 were also elevated in *Xiap*^*−/−*^ livers (Fig. S4A). CCL3 and CCL4 mediate granulocyte recruitment [27]. Therefore, CCL3 or CCL4 might have contributed to the depletion of *Xiap*^*−/−*^ splenic granulocytes, while enhancing numbers of granulocytes in the liver (Figs. 4D and S4A, B). Inhibition of RIP1, but not RIP2, suppressed IL-6, CCL3, or CCL4 levels in *Xiap*^*−/−*^ mice, and shifted granulocyte numbers closer to those found in TNF-treated control mice (Figs. 4B, D and S4A, B). We also explored the activation of cell death signaling in liver tissues and observed TNF stimulation dependent RIP1 phosphorylation and caspase-8 processing, and these modifications were both blocked by RIP1 inhibition (Fig. S4C). By contrast, we did not observe any differential processing of caspases 3, 1, or 11; GSDMD; IL-1β; or loss of c-IAP1/2 (Fig. S4C).

Therefore, TNF treatment of *Xiap*^*−/−*^ mice leads to RIP1 and caspase-8 activation that is accompanied by severe intestinal damage and depletion of granulocytes from spleen.

## Discussion

When XIAP was initially characterized, it represented the only endogenous mammalian direct inhibitor of caspases 3, 7 and 9 [1]. This discovery prompted a broad effort by numerous organizations to find an antagonist of XIAP that could be used to stimulate caspase activation and the death of cancer cells [24]. However, it soon became evident that XIAP antagonism alone does not cause sufficient cell death in tumors and that additional death triggers are needed [28]. In addition, mice lacking XIAP had no overt phenotype without any form of challenge [29, 30]. Nevertheless, subsequent studies of *Xiap*^*−/−*^ mice revealed a critical role for XIAP in bacterial infections, in the NOD2-RIP2 signaling pathway, and in the regulation of RIP3-dependent necroptosis [3, 5, 7, 8, 31]. It is clear now that XIAP contributes to multiple signaling pathways and is required for normal homeostasis in many organs, especially the intestines [4, 22, 23].

The relevance of XIAP for human health is exemplified by mutations that are associated with several inflammatory diseases [14, 15]. Initially, *XIAP* mutations were found in a relatively rare XLP-2 syndrome [12], but *XIAP* mutations are now correlated with a worse prognosis for GVHD patients and very early onset IBD [16–21]. These findings have prompted us to investigate novel treatment options for patients with deleterious *XIAP* mutations. Given that XIAP suppresses apoptotic and necroptotic cell death, we investigated whether blocking cell death dependent on the kinase activity of RIP1 might ameliorate the consequences of XIAP deficiency. Inhibition of RIP1 with GNE684 was effective at ameliorating TNF-induced intestinal damage in mice lacking XIAP, whereas inhibition of RIP2 was not. This result is consistent with the NOD2-RIP2 signaling pathway not being a major modulator of cell death signaling by TNF or LPS.

Several RIP1 inhibitors have been and are currently being tested in clinical trials for the treatment of diverse inflammatory and neurodegenerative diseases [32]. Trials with GSK2982772 have not benefited patients with rheumatoid arthritis, ulcerative colitis or psoriasis, but it is possible that patients with active RIP1 were not enrolled in these studies [32]. Our data suggest that patients with *XIAP* mutations and suffering from inflammatory conditions would likely have active RIP1. RIP1 activation is predicted to be associated with tissue damage and there is increasing evidence of tissue damage-associated inflammatory conditions in patients with *XIAP* mutations [16, 25]. Further study of RIP1 activation in *XIAP* mutant patients, especially in the intestinal tissues, may provide important insights and pave the way for the eventual treatment with RIP1 inhibitors in inflammatory bowel disease and related pathologies.

## Materials and methods

### Reagents and antibodies

Human recombinant TNF, BV6, GNE684, and GSK583 were all synthesized at Genentech. Emricasan was purchased from Selleck Chemicals (S7775), zVAD from ABclonal and LPS from InvivoGen (tlrl-3pelps). The primary antibodies used were directed against: RIP1 (610459, BD Biosciences), pRIP1 S166 (#31122, Cell Signaling Technology (CST)), RIP3 (#15828, CST), pRIP3 (#91702, CST), MLKL (#MABc604, Millipore), pMLKL (#37333, CST), FADD (#05-486, Millipore), RIP2 (#22763, Santa Cruz), XIAP (M044-3, MBL, 66800-1-Ig, Proteintech), c-IAP2 (Genentech), c-IAP1/2 (#3400, R&D), caspase-1(Genentech), caspase-3 (#9661, 9662, CST), caspase-7(#9491, CST), caspase-8 (#8592, 9429, 4927, CST), caspase-11(#14340, CST), GSDMD (#50928, CST), IL-1b (AF-401-NA, R&D), JNK(#9252, CST), pJNK (#4668, CST), p65(#8242, CST), p-p65(#3033, CST), IkBa (#9242, CST), p-IkBa (#2859, CST), p38 (#9212, CST), p-p38 (#9211, CST), ERK (#4695, CST), p-ERK (#4370, CST), actin (A3853, Sigma), GapDH (#2118, CST), HSP90 (#4877, CST).

### Cells, organoids, and viability assays

BMDMs were extracted from femur and tibia of adult mice. Cells were cultured for 6 days in DMEM High Glucose supplemented with 10% heat inactivated fetal bovine serum, 2 mM GlutaMAX (Gibco), 100 U/ml Penicillin and 100 μg/ml Streptomycin (Gibco), and 50 ng/ml recombinant murine M-CSF (Genentech). The cells were cultured on non-treated plates.

Organoids were developed from small intestines of WT and *XIAP*^*−/−*^ mice following procedures described previously [33], and using IntestiCult Organoid Growth mouse Medium (Stemcell Technologies). Organoid viability was assessed by MTT assay as described previously [34].

Cell death was analyzed using Incucyte ZOOM and S3 (Essen BioSciences) using Sytox Green nucleic acid stain (S7020, ThermoFisher). 200 μg/ml digitonin (Sigma Aldrich) was used to lyse all cells at the end of the assay and this value was used to normalize each measurement to the total amount of cells in a given culture vessel. Additional cell death assays were done using quantifying lactate dehydrogenase (LDH) release (G1780, Promega).

### Western blot analysis

For western blot analyses tissues were lysed in Triton or Urea buffers. Triton buffer: 1% Triton X-100, 25 mM Tris-HCl buffer (pH 7.5), 150 mM NaCl, 1 mM EDTA, Halt Protease and Phosphatase Inhibitor Cocktail (Thermo Scientific). Cells were lysed on ice for 30 min and centrifuged at 14,000 rpm for 10 min at 4 °C [35]. 6 M urea containing buffer: 20 mM Tris–HCl pH 7.5, 135 mM NaCl, 1.5 mM MgCl_2_, 1 mM EGTA, 1% Triton X-100, 6 M urea and Halt Protease and Phosphatase Inhibitor Cocktail (Thermo Scientific). Tissues were lysed for 30 min at RT and centrifuged at 14,000 rpm for 10 min at 16 °C. Lysates were resolved on SDS-PAGE and immunoblotted with the indicated antibodies.

### Mice for animal studies

*Xiap*^*−/−*^ mice were described previously [30]. All animals were dosed and monitored according to guidelines from the Institutional Animal Care and Use Committee (IACUC) on study protocols approved by the Laboratory Animal Resource Committee at Genentech. Whenever possible, littermates were used, and all animals were randomized during group allocation. Pathologists assessed the samples in a blinded fashion. All data were analyzed by appropriate statistical tools (listed with the description of different methods/models) and all experiments included control groups. All individuals participating in animal care and use were required to undergo training by the institution’s veterinary staff.

### TNF induced SIRS

Systemic inflammatory response syndrome (SIRS) was induced in male littermates by intravenous (iv) injection of mouse TNF (Genentech) alone or together with zVAD-FMK (10 mg/kg) (APExBIO). Mice were grouped according to genotypes and the studies were unblinded. Body temperature was monitored using a rectal probe and a digital thermometer. Mice were euthanized if their body temperature was below 25 °C or if severely lethargic. Statistical analyses were done using Student’s *t* test (body temperature) or Mantel–Cox (log rank; for comparison of survival curves) were performed using the GraphPad Prism software.

### LPS + Emricasan endotoxemia model

Littermates of both sexes were intraperitoneally injected with 20 mg/kg LPS (tlrl-3pelps, InvivoGen) and 2.5 mg/kg Emricasan (S7775, Selleck Chemicals). The mice were subsequently monitored for 8 h and body temperature was determined every 2 h using a rectal temperature probe.

### LPS + GalN liver injury model

Liver injury was induced in male mice by injecting them with LPS (700 mg/kg) and GalN (5 μg/kg) i.p. Serum was collected after 5 h, and ALT and AST were measured in a serum chemistry analyzer (Beckman Coulter AU480).

### Cytokine and chemokine detection

Sera of adult mice or cell supernatants were analyzed by Luminex (Bio-Plex Pro Mouse Cytokine 23-plex assay, Bio-Rad).

### Real-time quantitative PCR (RT-qPCR)

Total RNA was extracted from small intestines tissue samples using the RNeasy plus mini kit (QIAGEN) following manufactures instructions. An on-column DNase treatment was included. cDNA was generated from each RNA sample using a Taqman Gene Expression Cells-to-CT kit (Thermo Fisher Scientific). Gene expression assay for Tnf (Mm00443260_g1), Ccl2 (Mm00441242_m1), Birc3 (Mm01168413_m1), Nfkbia (Mm00477800_g1), Cxcl1 (Mm04207460_m1) and GAPDH (Mm99999915_g1) were from Thermo Fisher Scientific and for Icam1 (Mm.PT.58.43714327) was from IDT. All mRNA expression levels were normalized to GAPDH gene expression.

### Histology and immunohistochemistry (IHC)

Small intestinal histology was scored for villous atrophy (1, minimal brush border irregularity; 2, cobblestoning with minimal atrophy; 3, villous blunting and atrophy resulting in approximately 25-50% reduction in expected height; 4, villous blunting and atrophy with an estimated >50% reduction in height), crypt degeneration (1, rare pyknotic cells with no architectural loss; 2, individual pyknotic cells in approximately >25% of crypts with no architectural loss; 3, loss of crypt architecture in <25% of crypts; 4, less of crypt architecture in ≥25% of crypts), and inflammation (1, increased intravascular granulocytes; 2, aggregates of proprial granulocytes with no crypt separation; 3, multifocal granulocyte aggregates separate and elevate crypts; 4, extensive inflammatory infiltrates). Scores of these 3 parameters were scored in each intestinal segment available, and final scores represented the sum of all intestinal segments available per mouse. Goblet cells were assessed on PAS/Alcian blue stained sections by counting villous goblet cells in 10 villi/intestinal segment. Gr-1 immunohistochemistry was performed with a rat anti-Ly6G/Ly6C antibody (Pharmigen, clone RB6-8C5) at 2.5 µg/ml with Target retrieval, an anti-rat IgG rabbit linker antibody, and PowerVision polymer-based detection with Fast Red and hematoxylin counterstain. Splenic Gr-1 labeling was scored according to a 4-point matrix (1, low density of Gr-1 positive cells distributed throughout the red pulp; 2, mildly increased number of labeled cells in the red pulp; 3, moderately increased number of labeled cells in the red pulp with focal aggregation; 4, markedly increased number of labeled cells in the red pulp with prominent granulocyte aggregation). Hepatic Gr-1 labeling was quantitatively accessed on whole-slide image that were scanned on an Nanozoomer XR (Hamamatsu) using Matlab to quantify the tissue area and labeled cell number with the assessment reported as cell per square millimeter.

### Statistical analysis

Statistical analysis was performed using the GraphPad Prism software. The variance was assumed to be similar between the compared groups and that groups have normal distribution. Unpaired two-tailed *t* test (for two groups) or analysis of variance (for three or more groups) were used for all statistical significance except for comparison of survival curves that was analyzed by Mantel–Cox (log rank).

## Supplementary information

Supplemental material

Original Data File

completed checklist

## Data Availability

All data and materials reported in this study will be shared by the lead contact upon request.
