# Supplementary material for: XIAP deletion sensitizes mice to TNF-induced and RIP1-mediated death
Source: Cell Death Dis. 2023 Apr 11;14(4):262. doi: 10.1038/s41419-023-05793-1 (PMC10090100; doi:10.1038/s41419-023-05793-1)
Supplement: Supplementary file 1 — Supplemental material [file 41419_2023_5793_MOESM1_ESM.pdf]

Supplemental Figure Legends

Figure S1. Absence of XIAP enhances D-GalN/LPS-induced liver injury but does not affect LPS- or TNF-stimulated NF-κB and MAPK signaling.

A. BMDMs derived from WT or *Xiap*$^{-/-}$ mice were treated with LPS or LPS and zVAD (LZ). Percentage of dead BMDMs (Sytox Green positive cells) compared to positive control. Signal was measured every hour in the Incucyte with the indicated treatments. Measurements for control and LPS treatments are the same as shown in Figure 1A. (n=5 for WT non-treated control, n=6 for all others).

B. Necroptotic signaling in WT or *Xiap*$^{-/-}$ BMDMs. BMDMs were treated with LPS and Emricasan (Emric) for up to 8h. Western blots were performed with indicated antibodies.

C. Absence of XIAP does not affect LPS induced NF-κB and MAPK signaling. WT or *Xiap*$^{-/-}$ BMDMs were treated with LPS for up to 2h. Western blots were performed with indicated antibodies.

D. XIAP deficiency enhances D-GalN/LPS-induced liver injury. WT (n = 8) and *Xiap*$^{-/-}$ (n = 8) mice were injected with LPS (700 mg/kg) and GalN (5 μg/kg) ip. Serum levels of ALT and AST 5h post LPS/GalN are plotted.

E. Absence of XIAP does not affect TNF induced NF-κB and MAPK signaling. WT or *Xiap*$^{-/-}$ BMDMs were treated with TNF (20 ng/ml) for indicated times. Western blots were performed with indicated antibodies.

In panels A bars indicate median with standard error, and in panel D mean with standard deviation. ** indicates $p<0.01$ and *** $p<0.005$.

Figure S2. Antagonism of XIAP BIR2 domain sensitizes to TNF-induced SIRS.

A. WT mice were administered vehicle (n=3), TNF (500 μg/kg) (n=8), XIAP antagonist XB2m54 (m54) (n=5), or TNF and XB2m54 (n=12), while *Xiap*$^{-/-}$ mice (n=7) were injected with TNF. Survival (left) and body temperature (right) were monitored for 10h.

B. Serum levels of indicated cytokines from WT (n=6) and *Xiap*$^{-/-}$ (n=4) mice 3h post TNF and XB2m54 (only WT mice, n=6) treatment were analyzed by Luminex.

C. XB2m54 and TNF do not affect c-IAP1/2 or XIAP levels. Lysates of small intestines of WT mice treated with TNF and/or XB2m54 for 3h were examined by western blotting using indicated antibodies.

D. WT mice were administered vehicle (n=3), TNF (500 μg/kg) plus zVAD-FMK (10 mg/kg) (TZ) (n=9), XIAP antagonist XB2m54 (m54) (n=8), or TNF plus zVAD and XB2m54 (n=10), while *Xiap*$^{-/-}$ (n=5) mice were injected with TNF. Survival (left) and body temperature (right) were monitored for 10h.

In panels A and D bars indicate median with standard error, and in panels B mean with standard deviation. ** indicates p<0.01 and *** p<0.005.

Figure S3. XIAP deletion sensitizes BMDMs to RIP1-dependent TNF-induced death.

A. Cell death assay in BMDMs derived from WT or *Xiap*$^{-/-}$ mice. BMDMs were treated in triplicates with TNF (20 ng/ml) or TNF and zVAD (10 μM) in the absence or presence of RIP1 inhibitor GNE684 or RIP2 inhibitor GSK583 (each at 5 μM). Cell death was performed using LDH assay.

B. Necroptotic signaling in WT or *Xiap*$^{-/-}$ BMDMs. BMDMs were treated with TNF and zVAD in the absence or presence of RIP1 inhibitor GNE684 or RIP2 inhibitor GSK583. Western blots were performed with indicated antibodies.

C. Cell death assay in BMDMs derived from WT or *Xiap*$^{-/-}$ mice. BMDMs were treated in triplicates with LPS (100 ng/ml) or LPS and emricasan (5 μM) in the absence or presence of RIP1 inhibitor GNE684 (5 μM). Percent of cell death indicates Sytox Green positive cells compared to positive control measured in the Incucyte.

In panels A and C bars indicate median with standard error. Ns indicates no significance, *** p<0.005, **** p<0.001.

Figure S4. XIAP deficiency promotes TNF stimulated and RIP1 dependent granulocyte recruitment, and RIP1 and caspase-8 activation in the liver.

WT and *Xiap*$^{-/-}$ mice were treated with vehicle or TNF (500 μg/kg) for 3h in the absence or presence of RIP1 inhibitor GNE684 (50 mg/kg) or RIP2 inhibitor GSK583 (30 mg/kg).

A. RIP1 inhibitor reduces IL-6 and CCL3 levels in livers of TNF treated *Xiap*$^{-/-}$ mice.

B. RIP1 inhibition reduces recruitment of granulocytes to livers of TNF treated *Xiap*$^{-/-}$ mice. Gr-1 IHC was performed on sections of livers from WT or *Xiap*$^{-/-}$ mice treated with TNF (3h) and given RIP1 or RIP2 inhibitors. Quantification of Gr-1 positive cells depicted in the graph (left) with representative images (right). Size bars = 200 μm.

C. Protein levels and activation of signaling in livers of WT and *Xiap*$^{-/-}$ mice treated with TNF (3h) in the absence or presence of RIP1 or RIP2 inhibitors (n=3 mice per treatment). Western blots were performed with indicated antibodies.

In panels A and b bars indicate mean with standard deviation. Ns indicates no significance, * $p<0.05$, ** $p<0.01$.

Figure S1

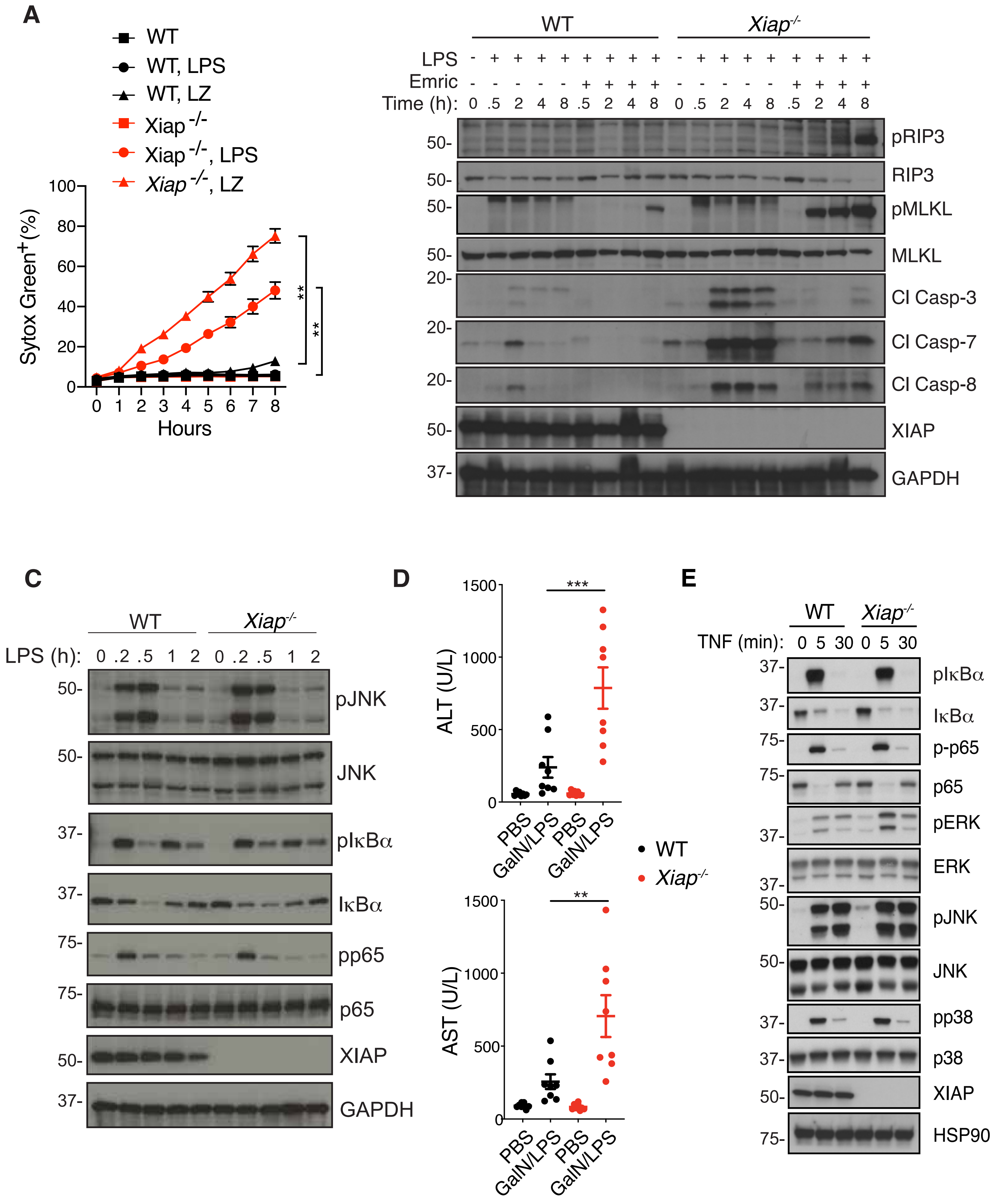

A
WT
WT, LPS
WT, LZ
Xiap -/-
Xiap -/-, LPS
Xiap -/-, LZ
Sytox Green+(%)
Hours
**
**
B
WT
Xiap-/-
LPS
Emric
Time (h):
pRIP3
RIP3
pMLKL
MLKL
Cl Casp-3
Cl Casp-7
Cl Casp-8
XIAP
GAPDH
C
WT
Xiap-/-
LPS (h):
pJNK
JNK
pIκBα
IκBα
pp65
p65
XIAP
GAPDH
D
ALT (U/L)
***
PBS
GalN/LPS
PBS
GalN/LPS
AST (U/L)
**
WT
Xiap-/-
E
WT
Xiap-/-
TNF (min):
pIκBα
IκBα
p-p65
p65
pERK
ERK
pJNK
JNK
pp38
p38
XIAP
HSP90

Figure S2

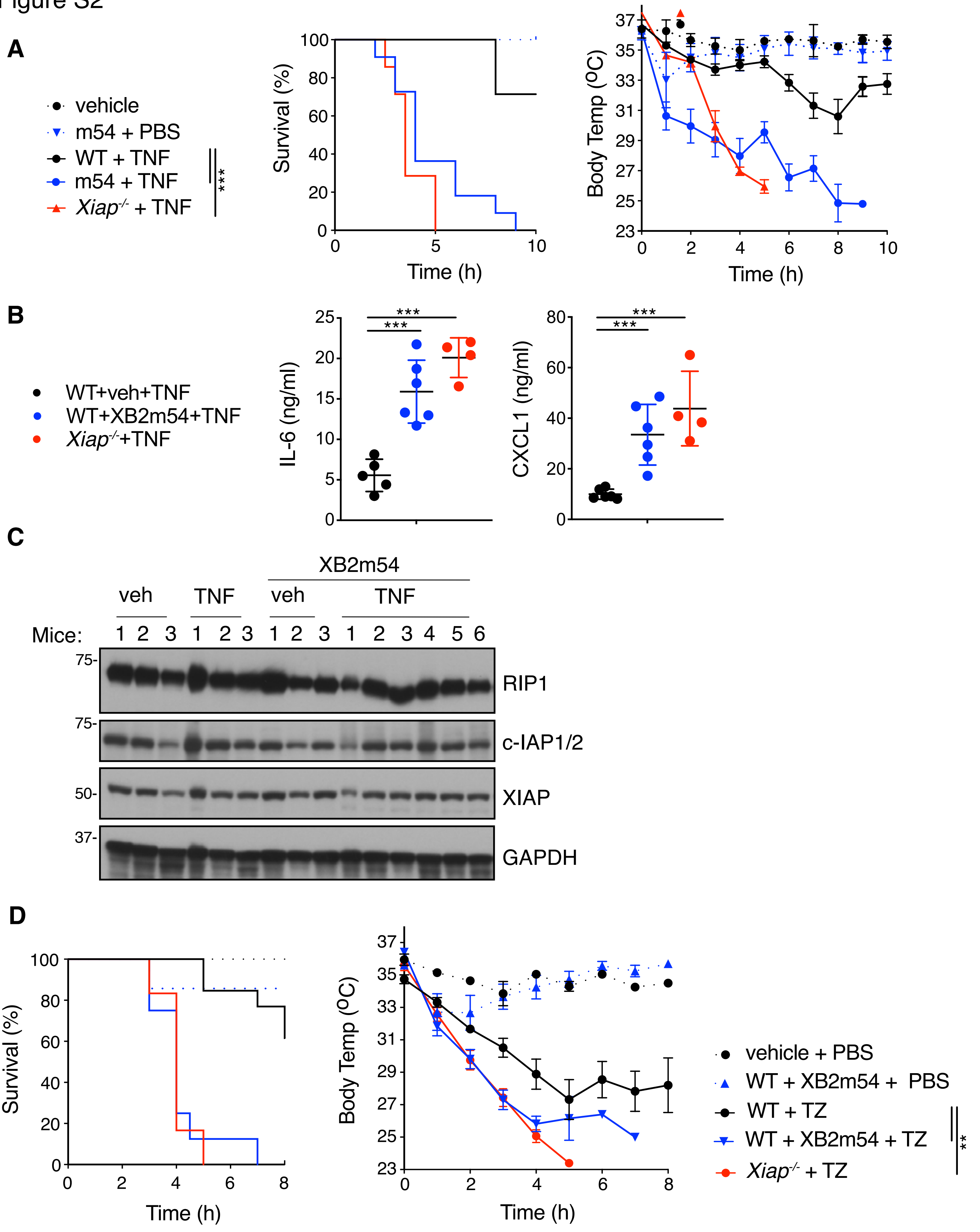
A
vehicle
m54 + PBS
WT + TNF
m54 + TNF
Xiap-/- + TNF
***
Survival (%)
Time (h)
Body Temp (oC)
Time (h)
B
WT+veh+TNF
WT+XB2m54+TNF
Xiap-/-+TNF
IL-6 (ng/ml)
CXCL1 (ng/ml)
***
***
C
XB2m54
veh
TNF
veh
TNF
Mice: 1 2 3 1 2 3 1 2 3 1 2 3 4 5 6
75-
RIP1
75-
c-IAP1/2
50-
XIAP
37-
GAPDH
D
Survival (%)
Time (h)
Body Temp (oC)
Time (h)
vehicle + PBS
WT + XB2m54 + PBS
WT + TZ
WT + XB2m54 + TZ
Xiap-/- + TZ
**

Figure S3

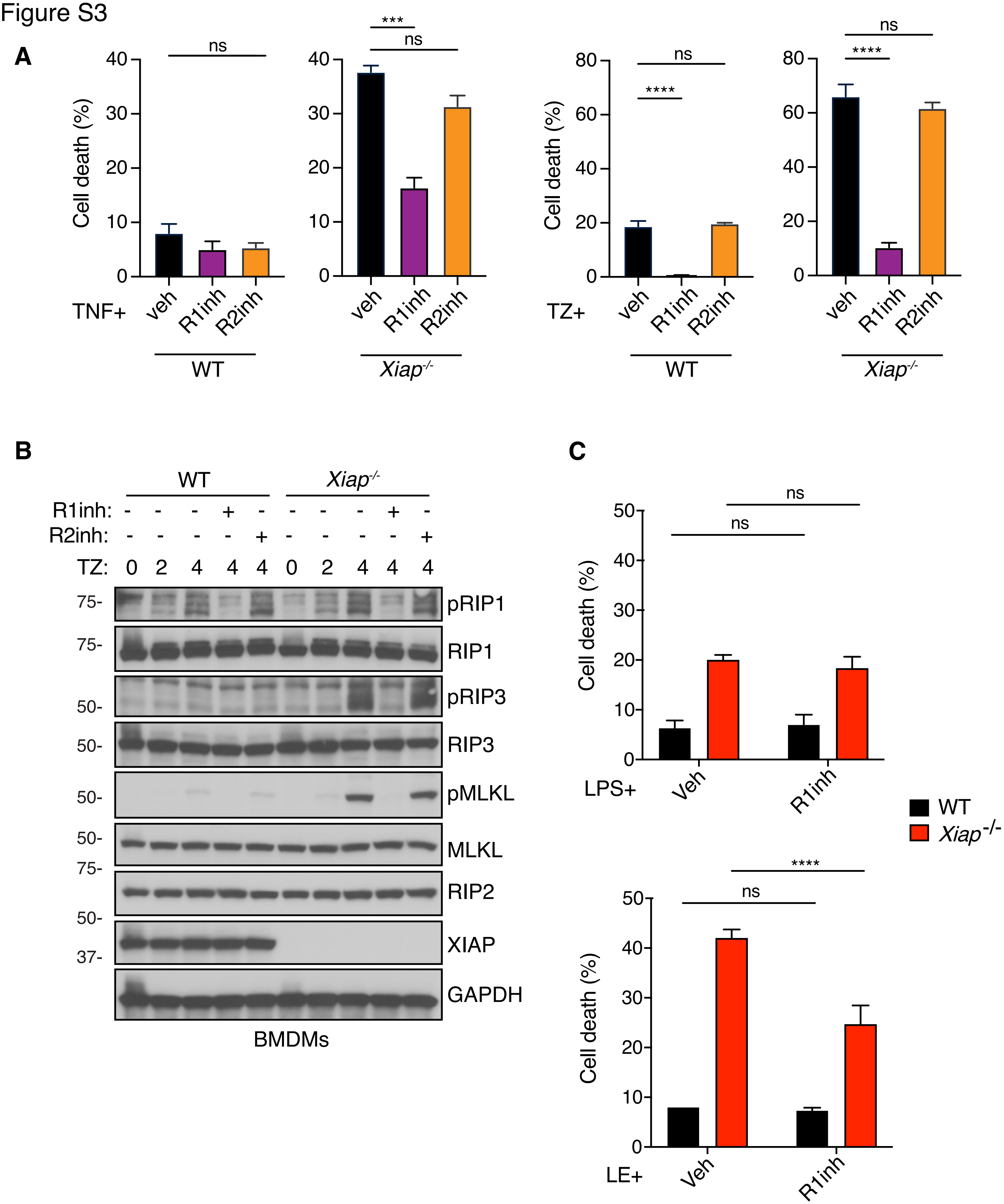
A
Cell death (%)
TNF+
veh
R1inh
R2inh
WT
Xiap-/-
ns
***
TZ+
****
B
WT
Xiap-/-
R1inh:
R2inh:
TZ: 0 2 4 4 4 0 2 4 4 4
75-
50-
37-
pRIP1
RIP1
pRIP3
RIP3
pMLKL
MLKL
RIP2
XIAP
GAPDH
BMDMs
C
LPS+
Veh
R1inh
WT
Xiap-/-
LE+

Figure S4

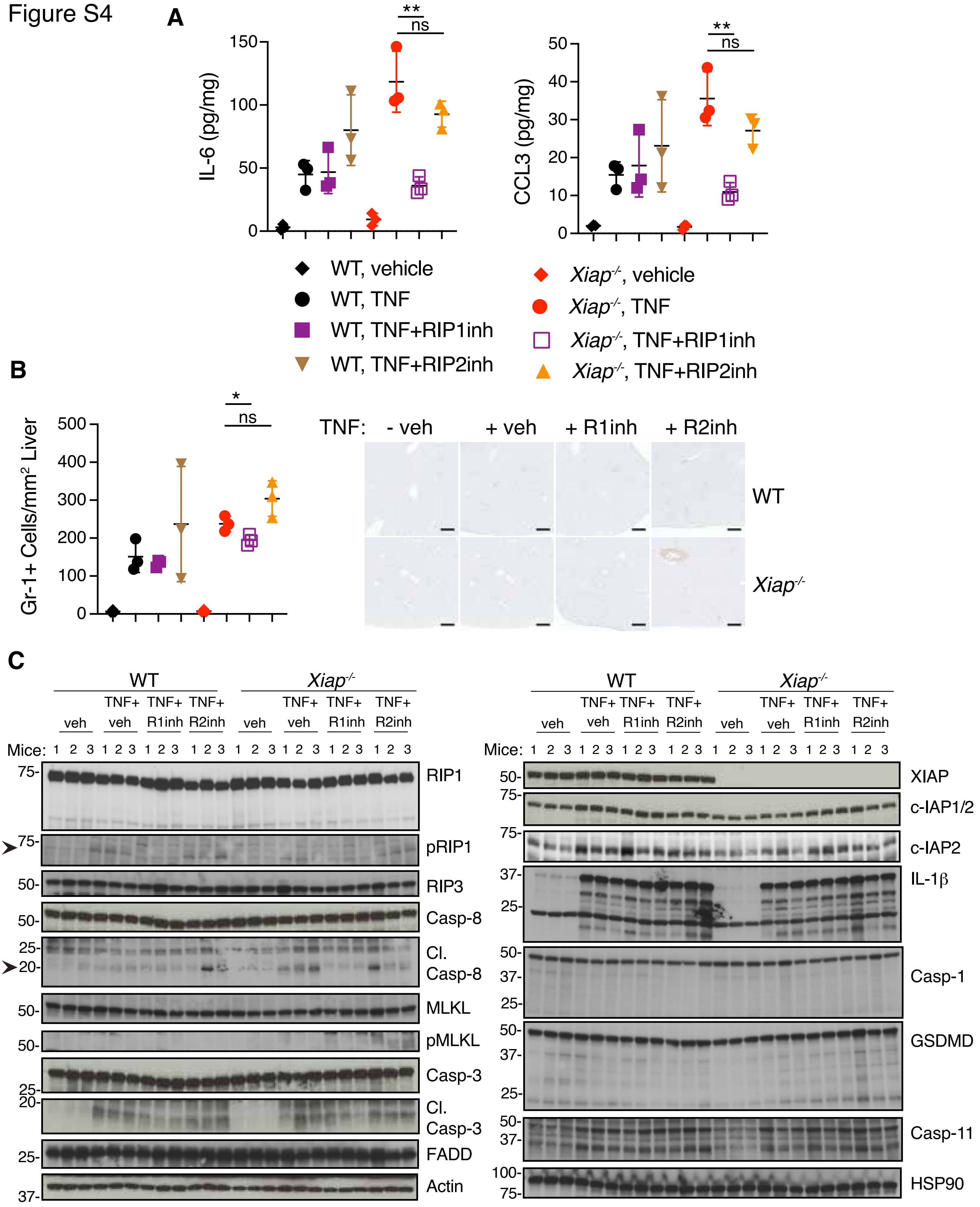

A
IL-6 (pg/mg)
150
100
50
0
**
ns
CCL3 (pg/mg)
50
40
30
20
10
0
WT, vehicle
WT, TNF
WT, TNF+RIP1inh
WT, TNF+RIP2inh
Xiap-/-, vehicle
Xiap-/-, TNF
Xiap-/-, TNF+RIP1inh
Xiap-/-, TNF+RIP2inh
B
Gr-1+ Cells/mm2 Liver
500
400
300
200
100
0
*
ns
TNF: - veh + veh + R1inh + R2inh
WT
Xiap-/-
C
WT
Xiap-/-
veh
TNF+ veh
TNF+ R1inh
TNF+ R2inh
Mice: 1 2 3 1 2 3 1 2 3 1 2 3 1 2 3 1 2 3 1 2 3 1 2 3
RIP1
pRIP1
RIP3
Casp-8
Cl. Casp-8
MLKL
pMLKL
Casp-3
Cl. Casp-3
FADD
Actin
XIAP
c-IAP1/2
c-IAP2
IL-1β
Casp-1
GSDMD
Casp-11
HSP90
