# Supplementary figures and images for: XIAP deletion sensitizes mice to TNF-induced and RIP1-mediated death
Source: Cell Death Dis. 2023 Apr 11;14(4):262. doi: 10.1038/s41419-023-05793-1 (PMC10090100; doi:10.1038/s41419-023-05793-1)

Fig S1B

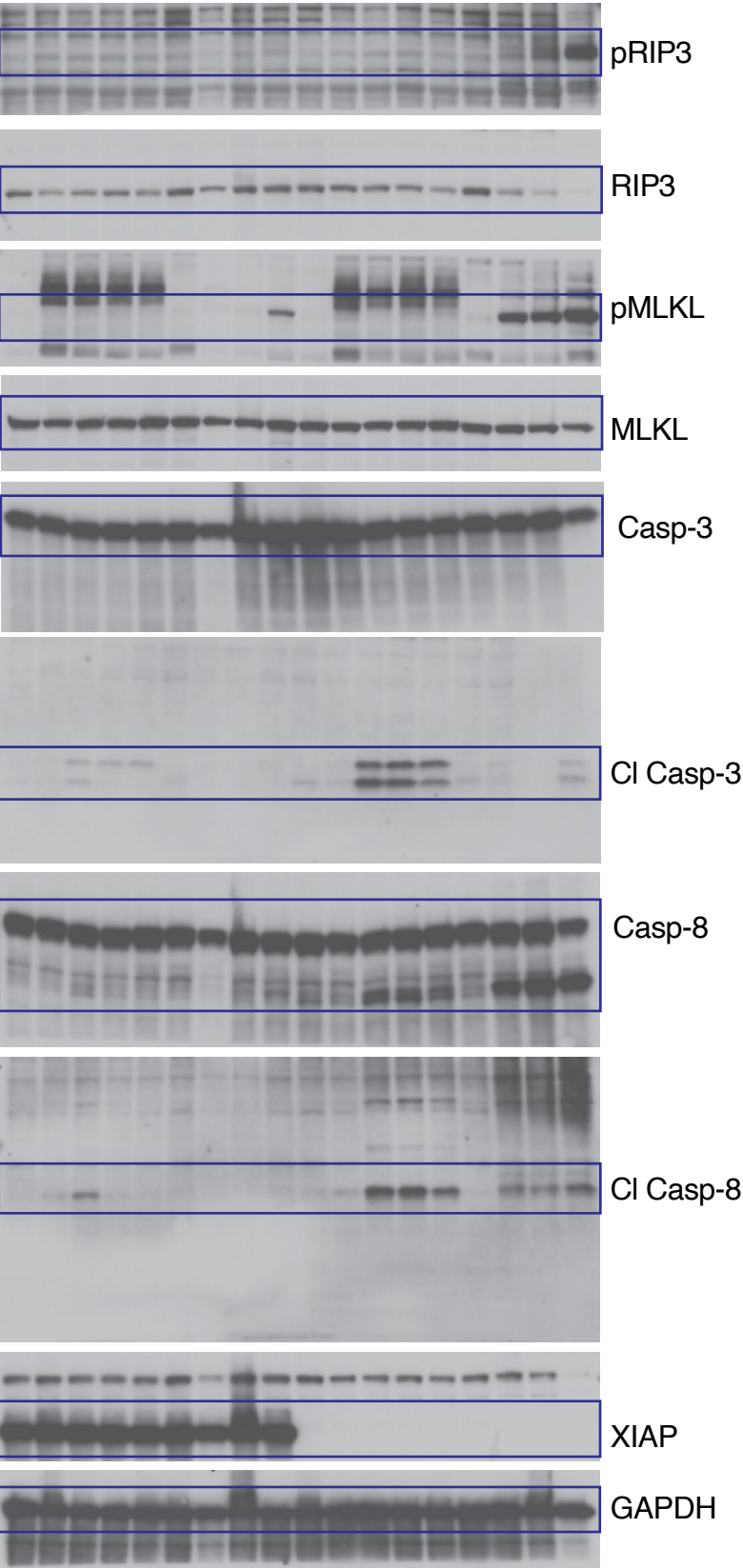

Fig S1C

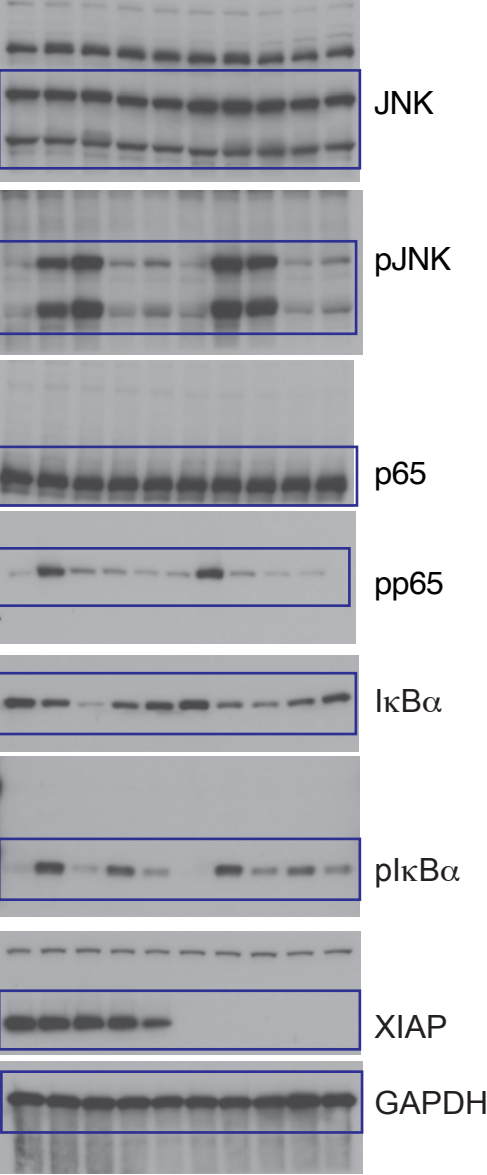

Fig S1E

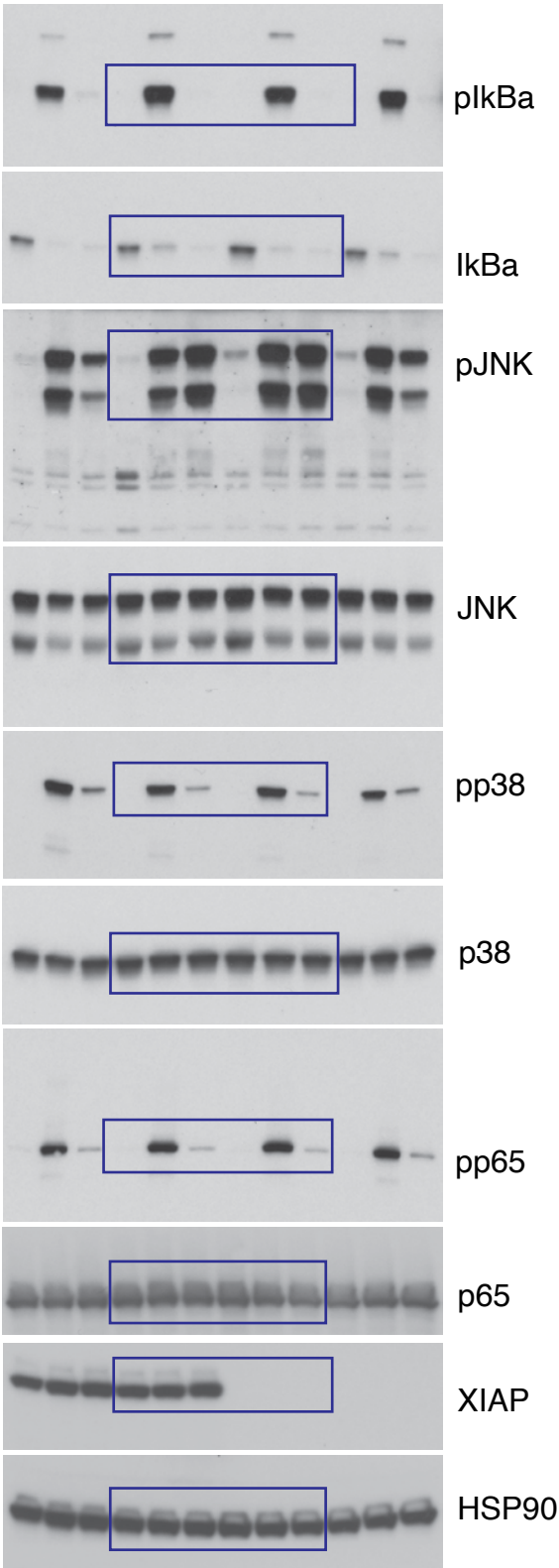

Fig S2C

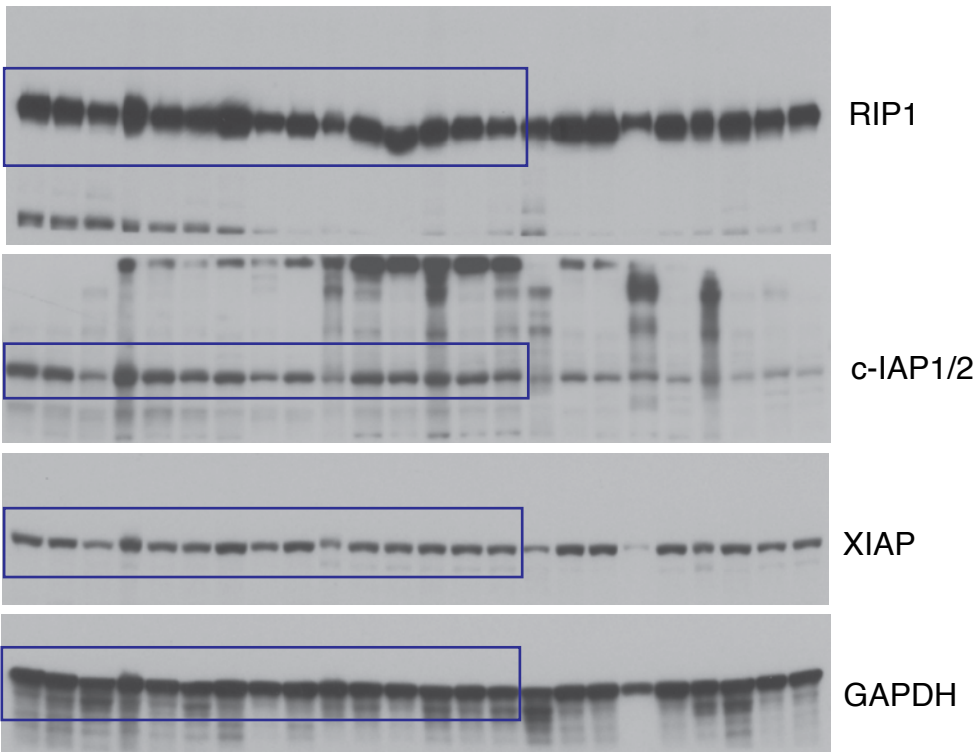

Fig 3E

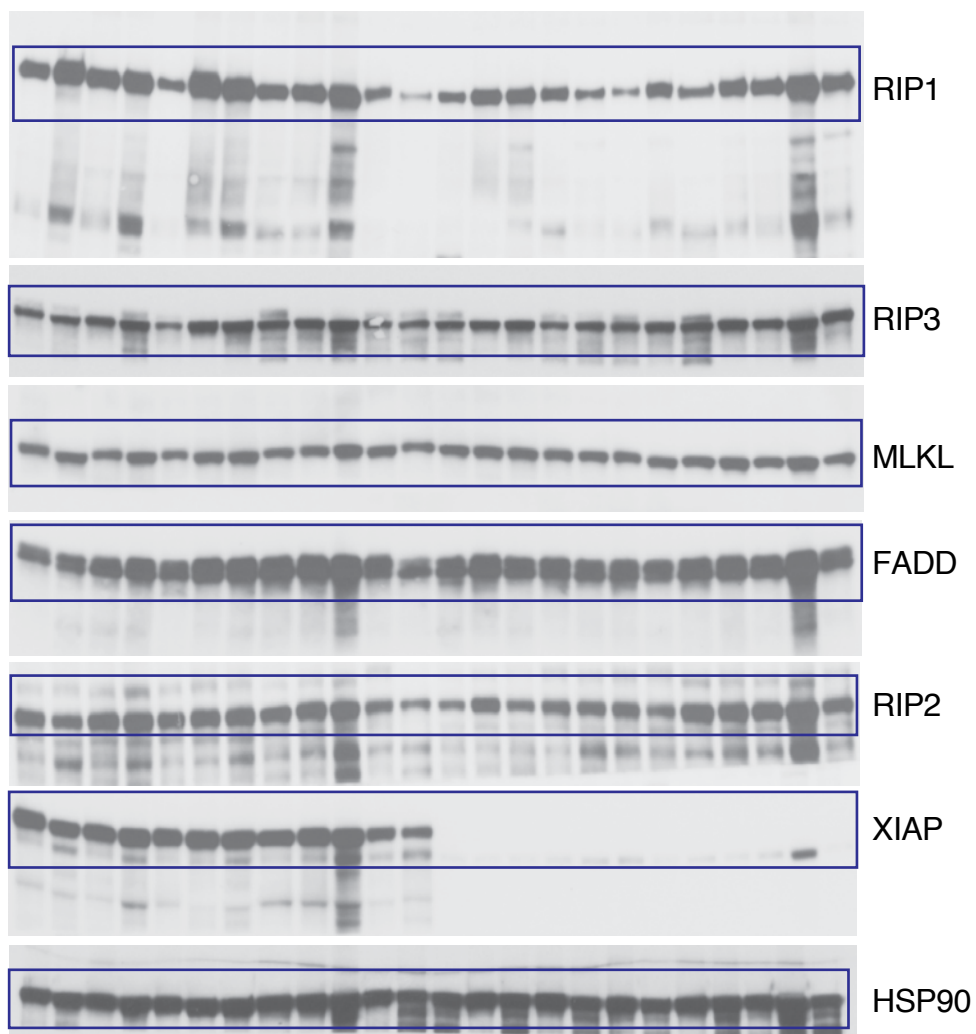

Fig S3B

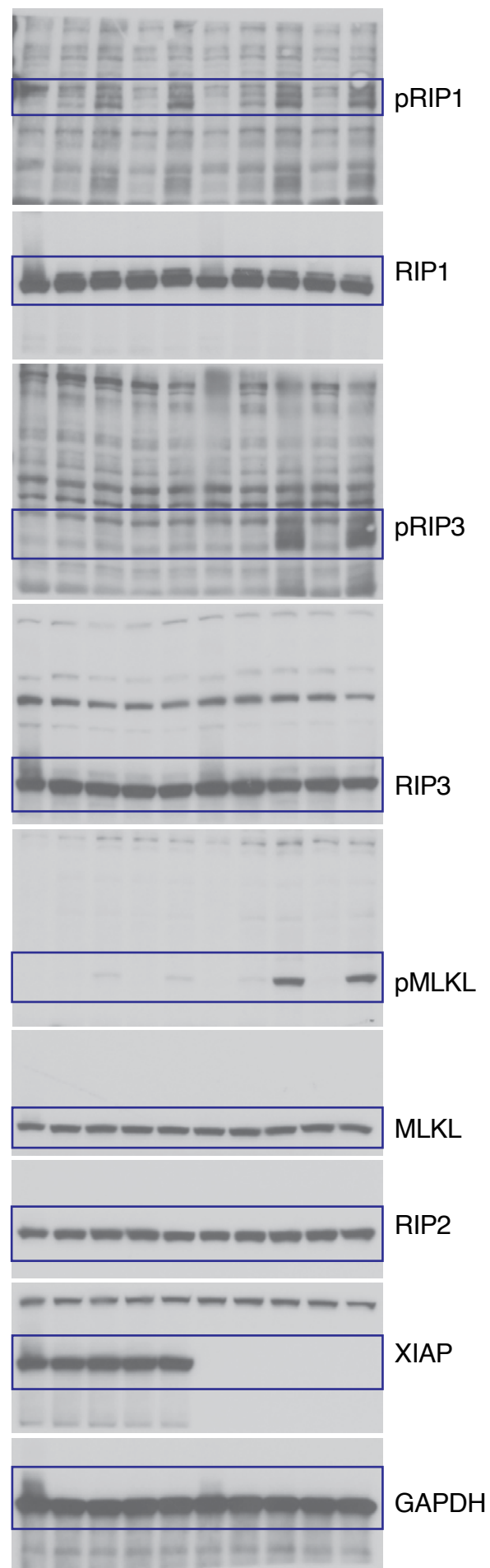

Fig S4C

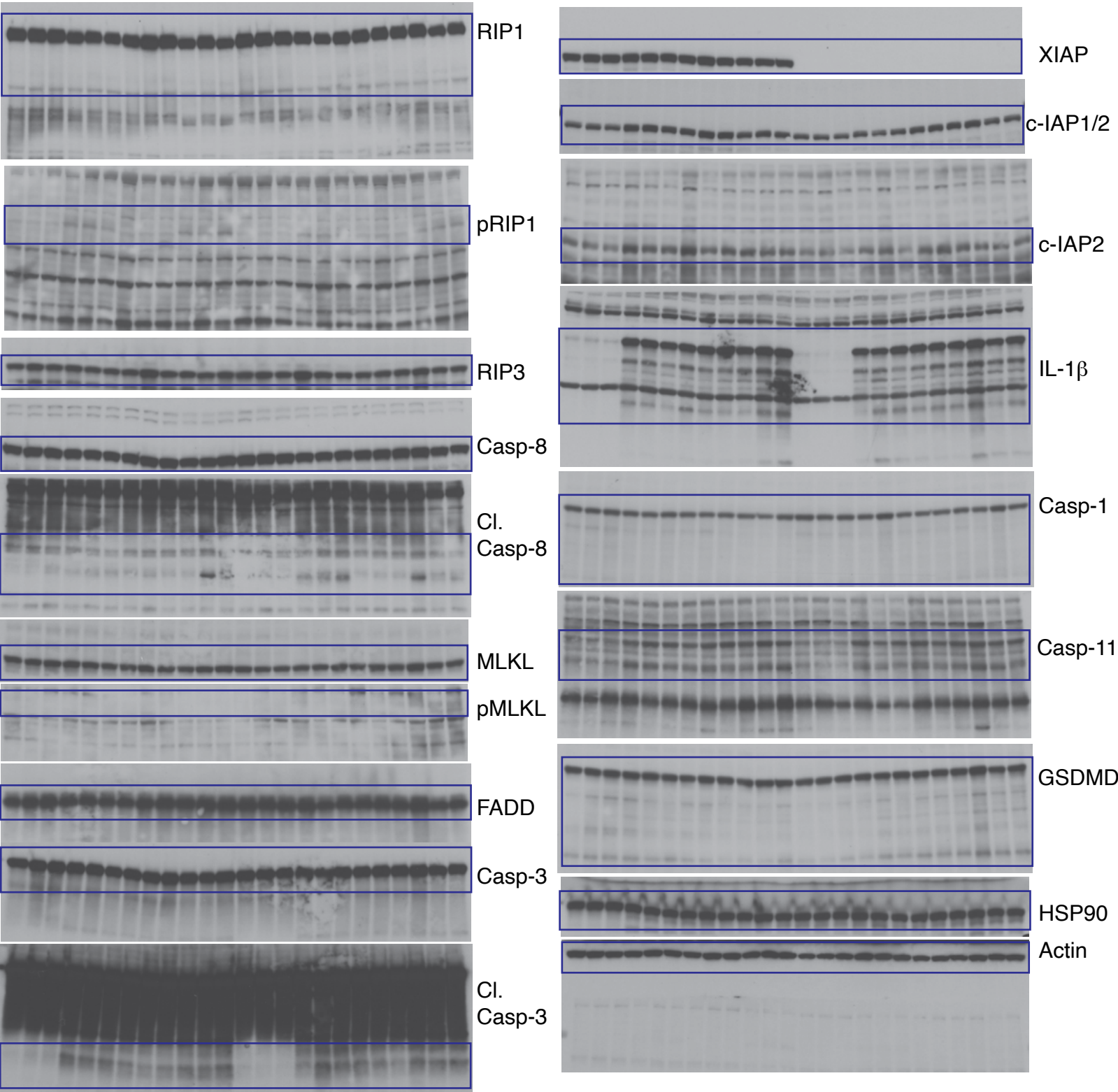

Supplement: Supplementary file 2 — Original Data File [file 41419_2023_5793_MOESM2_ESM.pdf]
